# The cost-efficacy of a healthy food box for managing hypertension within a native American population: a group randomized controlled trial

**DOI:** 10.1186/s13690-024-01274-9

**Published:** 2024-04-26

**Authors:** Austin Henderson, Robert Rosenman, Amber L. Fyfe-Johnson, Tori Taniguchi, Joy Standridge, Tyra Shackleford, Clemma J. Muller, Jason G Umans, Valarie Blue Bird Jernigan

**Affiliations:** 1 Institute for Research and Education to Advance Community Health, Elson S. Floyd College of Medicine, Washington State University, Seattle, Washington United States of America; 2 School of Economic Sciences, Washington State University, Pullman, Washington United States of America; 3 Center for Indigenous Health Research and Policy, Oklahoma State University Center for Health Sciences, Stillwater, Oklahoma United States of America; 4 Nutrition Services Department, Chickasaw Nation, Ada, Oklahoma United States of America; 5 Hyattsville MD and Georgetown-Howard Universities Center for Clinical and Translational Science, MedStar Health Research Institute, Washington DC, United States of America

**Keywords:** Hypertension, Native health, Food Sovereignty, Nutrition, Cost-effectiveness

## Abstract

**Background:**

Dietary interventions are used for the treatment of hypertension. We evaluated the cost-efficacy of delivering boxes of healthy, culturally tailored foods and checks that can only be spent on produce in a Native American population.

**Methods:**

We conducted a group randomized controlled trial from 2018 to 2020 with *N* = 2 treatment counties and *N* = 2 control counties and a total of *N* = 160 Native American adults with baseline stage 1 or stage 2 hypertension. Participants in the intervention group received monthly boxes of food that adheres to the Dietary Approaches to Stop Hypertension diet as well as checks that could only be spent on produce for 6 months. We measured blood pressure and quality of life at baseline and at a 6-month follow-up in both intervention and control groups. We used ordered logistic regression to estimate the effect of treatment on probability of blood pressure improvements. We then conducted a cost-efficacy analysis.

**Results:**

We found that treatment was effective in reducing blood pressure in women with stage 1 hypertension at baseline. Based on this finding, we also estimate that this intervention satisfies normative cost-effectiveness thresholds, even when lifetime treatment is needed to preserve the impact, so long as treatment is only continued in those who respond to treatment.

**Conclusions:**

Direct delivery of healthy foods and checks that can only be spent on produce are a potentially cost-effective intervention for the management of hypertension among Native American women with stage 1 hypertension. Further research is needed to understand why we found an impact only for this group.

**Supplementary Information:**

The online version contains supplementary material available at 10.1186/s13690-024-01274-9.

**Table:** 

Text box 1. Contributions to the literature
**•** This study is the first randomized controlled trial of a dietary intervention for hypertension management in a specifically Native American population.
**•** A Tribally-administered program which directly delivered healthy and culturally tailored foods to Native American adults with hypertension was effective in lowering blood pressure in women with stage 1 hypertension at baseline.
**•** The treatment is cost-effective in women with stage 1 hypertension even if it must be administered for the lifetime of recipients, under a set of plausible assumptions.

## Introduction

Cardiovascular disease (CVD) and related risk factors, including hypertension and obesity, is more prevalent in Native Americans than in all-race populations, a fact that has persisted for decades [1–4]. Accordingly, public health strategies which can meaningfully reduce hypertension in this population have value to individuals, tribes, and the healthcare systems supporting them. While there has been substantial research into, investment in, and application of pharmacological strategies for addressing hypertension (including studies focusing on Native Americans, e.g., Howard et al., [5]), there is still a dearth of medical knowledge of efficacious non-pharmacological interventions to address hypertension among Native American populations. Because many of the risk factors for CVD have been found to be associated with poor food environments [6], a situation facing many Native Americans [7], nutrition-based programs are of special interest.

Building upon a substantial literature on dietary interventions to improve blood pressure (BP) [8–15] the Chickasaw Healthy Eating Environment Research Study (CHEERS) was a group randomized controlled trial (RCT) in which hypertensive Native American adults living in the Chickasaw Nation were given monthly boxes of healthy, culturally tailored foods, “Fresh Checks” that could be spent only on fruits and vegetables, as well as instructions and guidance for preparation. The study was conducted from 2018 to 2020. Participants were followed for 6 months. A comprehensive questionnaire collected data on blood pressure, weight, diet, physical activity, and medication at both baseline and follow-up.

In this paper, we conduct an economic analysis of CHEERS building upon two key aspects of hypertension and hypertension management. First, the health risks of hypertension increase with hypertension severity in a log-linear fashion [16], suggesting a heightened importance of addressing hypertension in those with the highest severity. Second, prescribed treatment usually depends on the category of hypertension [17]. Blood pressure categories are defined according to American College of Cardiology / American Heart Association 2017 guidelines, with normal blood pressure defined as SBP < 120 mm Hg and DBP < 80 mm Hg, elevated blood pressure as SBP of 120–129 mm Hg and DBP < 80 mm Hg, stage 1 hypertension as SBP of 130–139 mm Hg or DBP of 80–89 mm Hg, and stage 2 hypertension as SBP > = 140 mm Hg or DBP > = 90 mm Hg.

Thus, we examine the influence of our intervention conditional on baseline hypertension category. Because recent research indicates that women would have greater cardiovascular benefits from decreases in BP, we also analyze our results based on the sex of participants [18]. 

## Methods

### Sample and study

This study was conducted in partnership with the Chickasaw Nation, a federally recognized Native American Tribe, which shares geography with the state of Oklahoma. Chickasaw Nation, the thirteenth largest federally recognized tribe in the U.S, is in southcentral Oklahoma with a reservation area, or jurisdictional territory as it is now called, consisting of 7,648 square miles and encompassing 13 Oklahoma counties. According to the most recent U.S. Census Bureau data, the Chickasaw Nation has a Native American population of 23,158 and a total population of 307,640 [19]. In 2010 the Center for Indigenous Health Research and Policy at the Oklahoma State University Center for Health Sciences (OSU-CHS) partnered with the Chickasaw Nation on a community-based participatory research study to improve the food environment for Native Americans residing in rural Oklahoma. That study increased healthy food choices in tribal convenience stores [20]. This (CHEERS) study builds upon and expands this long standing and successful partnership between OSU-CHS and the Chickasaw Nation.

Citizens of the Chickasaw Nation experience substantial barriers to healthy eating and report a high prevalence of food insecurity [21, 22]. They also report disproportionate cardiovascular disease risk factors including hypertension [22]. 

Eligible participants needed to be 18 years of age or older, Native American, and residing in Pontotoc, Johnston, Carter, or Murray counties in Oklahoma, have no plans to move out of the community within the next year, a prior clinical hypertension diagnosis (self-reported, not verified) or a measured systolic BP ≥ 130 mmHg on two separate days, and not be currently pregnant or < 6 weeks postpartum. Only participants with stage 1 or 2 hypertension at baseline are included in this analysis. Despite the enrollment criterion of a measured systolic BP > 130, 28 participants with measurements below this threshold were enrolled in the project. They were excluded from this analysis, as the treatment was not intended for people without stage 1 or greater hypertension.

Participants were recruited at health fairs, community events, via the tribal newspaper, and in the lobby of health clinics within the intervention (Pontotoc and Johnston counties) and control communities (Carter and Murray counties). To maximize recruitment efforts, CHEERS study information was printed in newsletters and advertisements, emailed to tribal citizens, and posted on bulletin boards at various establishments. Individuals were screened to be sure they met eligibility requirements. Once screened, study staff made an appointment for the individual to come to a local Chickasaw Nation facility in their community where the individual provided informed consent and baseline measures (i.e., height, weight, BP, and survey data) were collected.

Participation in the study was incentivized, and participants were given a $20 Visa or Mastercard gift card for completing each of biometrics, surveys, and dietary recalls at baseline and follow-up, for a maximum total of $120 per individual. Each survey was designed to take approximately 90–120 min to complete.

A total of 268 eligible individuals provided informed consent for this study, of whom *N* = 35 individuals were excluded due to incomplete BP baseline data, and *N* = 28 were excluded due to not having stage 1 or stage 2 hypertension at baseline. *N* = 26 did not complete follow-up BP measurements, and *N* = 19 later initiated withdrawal from the study and all data was excluded. Hence, we had a sample of *N* = 160 used in this analysis (*N* = 110 treatment; *N* = 50 control). BP measurements were defined by the average of the 2nd and 3rd of 3 measurements taken at both baseline and follow-up. Surveys asked questions on demographics, exercise, diet, and medications using validated instruments.

Group randomization by county was used in this study. Enrolled participants in Pontotoc and Johnston counties were assigned to the intervention group, while participants from Carter and Murray counties were in the control group. For six months, those in intervention counties received monthly home delivery of food boxes with Dietary Approaches to Stop Hypertension (DASH) specific foods, vouchers restricted for use only on fruits and vegetables (“Fresh Checks”), free tribal gym membership, a Fitbit, and access to AYA, a culturally based mobile walking app. The food boxes were valued at $55 each and the vouchers were for $20 worth of fresh produce. See Supplementary Materials S.1 for more details on Fresh Checks and what was included in the food boxes.

The DASH diet consists of foods such as poultry, fish, fruits, vegetables, whole grains, and low-fat dairy [23]. Other studies have found detectable changes in blood pressure from adhering to DASH diets in more controlled settings for one month (e.g. Sacks et al., [23] ). The six-month timespan of our study was chosen to balance considerations including the fact that our intervention was less comprehensive (for instance, not covering all meals), which means it may take longer to observe treatment effects, and the need to collect data quickly to reduce attrition.

Participants in control counties received free tribal gym membership, a Fitbit, and access to the AYA app. Therefore the comparison is between those who received the home delivery of food boxes and Fresh Checks and those who did not, and the analysis is of the marginal impact of the food boxes and Fresh Checks over receiving just the gym membership, Fitbit, and AYA.

Memoranda of understanding between the Chickasaw Nation, OSU-CHS, and Washington State University (WSU), including provisions pertaining to financial and research activities, were established before launching the study. The Chickasaw Nation Institutional Review Board (IRB) oversaw all aspects of the partnership. The study was reviewed and approved by OSU-CHS, WSU, and Chickasaw Nation institutional review boards. Tribal approval was obtained prior to submitting this manuscript for publication.

### Analytic approach

The goal of our analysis is to identify the conditions under which the intervention is cost-effective. We first check for intervention effectiveness, examining subgroups of participants divided by sex and baseline blood pressure category. We have three reasons for examining these subgroups. First, the benefits in terms of life expectancy of a given improvement in BP are dependent upon the baseline BP and sex of an individual. Second, identifying specific subgroups that benefit from treatment allows the targeting of limited resources to those groups. Third, these subgroups are easily identified as part of normal clinical practice in hypertension management. Once we identify the groups for whom the CHEERS treatment is effective, we then proceed with our cost-effectiveness analysis.

Because the CHEERS nutrition intervention was in *addition* to whatever care study participants were already receiving, there is no measured cost saving from reduced drug use or medical intervention. Instead, we assessed potential gains in life expectancy and quality of life due to incremental BP control associated with the CHEERS intervention, and then use these gains in a cost-effectiveness analysis. The primary outcome of the CHEERS intervention, defined prior to conducting the study, was systolic BP (SBP). In this paper, to facilitate translating changes in BP to changes in life expectancy, we analyze the related but separate concept of crossing specific BP thresholds.

As a reference for the life expectancy gains from a given improvement in BP we use Sesso et al., [24], who used data from a prospective dataset of 57,573 individuals to generate a Markov chain model which estimated changes in life expectancy for different magnitudes of BP reductions for various baseline levels and comorbidities. Our analysis used ordered logistic regression to estimate the average marginal effect of treatment on achieving the specific threshold BP changes they model. This gives us a conservative measure of the change in life expectancy that the treatment produces because improvements in BP are only considered to produce gains in life expectancy equivalent to the largest BP threshold crossed, e.g., an improvement of 15/9 mmHg is treated as an improvement of 13/8 mmHg. Gains in expected life years are then combined with changes in the quality of life, as measured with the EQ-5D-5 L [25], to produce estimates of changes in Quality Adjusted Life Years (QALYs). The EQ-5D-5 L is a five-item survey instrument which assesses quality of life on the dimensions of mobility, self-care, usual activities, pain/discomfort, and anxiety/depression. Responses to EQ-5D-5 L questions were converted to a utility index score using the 2020 Pickard value set version 2.1. It is on the changes of QALYs that we performed our cost-effectiveness analysis.

### Statistical analysis

Due to the group-level randomization of this trial, intra-cluster correlation (ICC) could be a serious concern for our estimates. However, we estimated an ICC of < 0.01 in SBP change, indicating a negligible level of correlation between observations at each site. Thus, we proceeded with our analysis at the individual level.

Sesso et al. estimated the life expectancy benefits of antihypertensive treatment, based on both systolic and diastolic blood pressure reduction with selected BP reductions which they argue are attainable and expected in a clinical setting. We used an ordered, multinomial logistic regression with the categories “BP fell by XX/YY mmHg or greater”, “BP neither fell nor gained by XX/YY mmHg or greater”, and “BP rose by XX/YY mmHg or greater” where the XX/YY are the Sesso et al. thresholds of 7/5, 13/8 and 20/13. We assume people who experience an increase in BP suffer life-expectancy losses that mirror life-expectancy gains from those who experience a decrease in BP, hence we account for this offset. All participants with a drop or gain of *at least* the stated threshold were put in the appropriate category. We did separate regressions for each sex and starting hypertension stage, and as no males with stage 1 hypertension, either control or treatment, had a change, up or down, in BP of 20/13 mmHg or more, we had 11 ordered logistic regressions of the form:

$$Y_{i} = \beta_{1}T_{i} + \beta_{2}X_{i}| Ci, Si$$

where Y_i_ is the individual’s outcome compared to a change of XX/YY mmHg, T indicates treatment, and X is a vector of covariates including age, BMI, and baseline SBP. C is initial BP category and S is sex. Robust standard errors were used.

## Results

### Comparison of groups

Descriptive statistics for the treatment and control groups at baseline are presented in Table 1. Treatment groups were well-balanced across all analytically important variables, including sex, age, and baseline blood pressure. There were no statistically significant differences in any presented variables.

**Table 1:** Baseline demographic and biometric characteristics of participants in a 2018–2020 study of hypertension management in Oklahoma

	Treatment Group			
	Control *N* = 50		Treatment *N* = 110	
**Sex**				
Female	33	66.0%	69	62.7%
Male	17	34.0%	41	37.3%
**Highest level of education completed**				
Highschool or less	18	36.0%	24	22.0%
Technical/vocational degree or Associate’s degree	8	16.0%	18	16.5%
Some college	14	28.0%	35	32.1%
College graduate (Bachelor’s degree) or post graduate degree	10	20.0%	32	29.4%
**Household Income**				
<=$15,000	2	4.7%	6	6.2%
$15,001 - $35,000	15	34.9%	28	29.2%
>=$35,001	26	60.5%	62	64.6%
**What is your current marital status?**				
Married	24	48.0%	47	43.1%
Partner/significant other but not married	5	10.0%	10	9.2%
Widowed and not remarried	3	6.0%	11	10.1%
Divorced and not remarried	11	22.0%	28	25.7%
Separated	2	4.0%	5	4.6%
Never married	5	10.0%	8	7.3%
**Employed**	47	94.0%	97	89.0%
**Moderate or vigorous physical activity in last week**	39	78.0%	90	81.8%
**Cigarette smoking frequency**				
Everyday	3	37.5%	14	26.9%
Some days	1	12.5%	12	23.1%
Never	4	50.0%	26	50.0%
**Been told by a medical person they have diabetes**	14	28.0%	34	32.4%
**Currently taking blood pressure medication**	33	76.7%	88	75.9%
**Baseline blood pressure category**				
Stage 1 hypertension	20	40.0%	44	40.0%
Stage 2 hypertension	30	60.0%	66	60.0%
**Baseline systolic blood pressure**	141.3	(14.0)	141.6	(11.5)
**Baseline diastolic blood pressure**	82.9	(11.5)	85.3	(9.3)
**Body mass index**	34.6	(8.5)	36.2	(10.1)
**Age in years**	49.6	(13.0)	49.1	(13.5)
**Days in the last week walked at least 10 min**	4.3	(2.5)	4.0	(2.6)

We present the mean of continuous variables with standard errors in parentheses

Table 2 summarizes the frequency and percent of individuals who dropped, gained, or neither the BP thresholds we used, separated by sex and baseline hypertension category.

**Table 2:** Blood pressure changes of participants in a 2018–2020 study of hypertension management in Oklahoma. Frequency and percent of individuals who dropped, gained, or neither specified blood pressure thresholds, separated by baseline ACA blood pressure category, sex, and treatment group

	Dropped or Gained 7/5 mmHg											
	Dropped				No change				Gained			
Starting BP Category	Control		Treatment		Control		Treatment		Control		Treatment	
**Female**												
Stage 1 hypertension	2	15.4%	9	42.9%	10	76.9%	10	47.6%	1	7.7%	2	9.5%
Stage 2 hypertension	9	45.0%	26	55.3%	10	50.0%	20	42.6%	1	5.0%	1	2.1%
**Male**												
Stage 1 hypertension	0	.	3	13.6%	5	71.4%	15	68.2%	2	28.6%	4	18.2%
Stage 2 hypertension	4	40.0%	8	42.1%	4	40.0%	10	52.6%	2	20.0%	1	5.3%
	**Dropped or Gained 13/8 mmHg**											
	Dropped				No change				Gained			
**Starting BP Category**	Control		Treatment		Control		Treatment		Control		Treatment	
**Female**												
Stage 1 hypertension	.		4	19.0%	12	92.3%	17	81.0%	1	7.7%	-	
Stage 2 hypertension	6	30.0%	8	17.0%	13	65.0%	38	80.9%	1	5.0%	1	2.1%
**Male**												
Stage 1 hypertension	.		.		7	100.0%	21	95.5%	.		1	4.5%
Stage 2 hypertension	3	30.0%	4	21.1%	7	70.0%	15	78.9%	.		.	
	**Dropped or Gained 20/13 mmHg**											
	Dropped				No change				Gained			
**Starting BP Category**	Control		Treatment		Control		Treatment		Control		Treatment	
**Female**												
Stage 1 hypertension	.		3	14.3%	13	100.0%	18	85.7%	.		.	
Stage 2 hypertension	5	25.0%	5	10.6%	14	70.0%	42	89.4%	1	5.0%	.	
**Male**												
Stage 1 hypertension	.		.		7	100.0%	22	100.0%	.		.	
Stage 2 hypertension	3	30.0%	2	10.5%	7	70.0%	17	89.5%	.		.	

Women in the treatment group with baseline stage 1 hypertension had a 37.5pp higher probability of dropping by 7/5 mmHg than those in the control group, while treated men with baseline stage 1 hypertension had a 13.6% higher probability of dropping by 7/5 mmHg than those in the control group. Similarly, both treated men and women with baseline stage 2 hypertension had a slightly higher probability of dropping by 7/5 mmHg than those in the control group (men 2.1%; women 8.3%).

The results differ when examining the larger thresholds. Treated women with baseline stage 1 hypertension had a 19.0pp higher probability of dropping by 13/8 mmHg and 14.3pp higher probability of dropping by 20/13 mmHg, but treated women with baseline stage 2 hypertension had a 13pp and 14.4pp *lower* probability to drop by 13/8 mmHg and 20/13 mmHg respectively than control women. There was only 1 man with baseline stage 1 hypertension who had a 13/8 mmHg movement, but treated men with baseline stage 2 hypertension had a 9.9pp and 29.5pp lower probability of dropping by 13/8 mmHg and 20/13 mmHg respectively.

### Average marginal treatment effects

Table 3 reports the average marginal treatment effect from the ordered logistic regressions for each sex, baseline hypertension stage, and threshold change. We note that the one female with Stage 2 hypertension who gained 13/8 mmHg was missing a covariate so was not included in the regression analysis.

**Table 3:** Average marginal treatment effects on the probability of dropping, gaining, or neither specific blood pressure thresholds for participants in a 2018–2020 study of hypertension management in Oklahoma

	Baseline Stage 2 Hypertension		Baseline Stage 1 Hypertension	
	**Female N = 57**	**Male N = 29**	**Female N = 35**	**Male N = 29**
	**Dropped or Gained 7/5 mmHg**		**Dropped or Gained 7/5 mmHg**	
**Treatment vs. Control**				
**Dropped**	0.06	0.02	0.14	0.08
	(−0.20, 0.32)	(−0.36, 0.41)	(−0.15, 0.43)	(−0.07, 0.23)
**No Change**	−0.06	−0.02	−0.08	0.11
	(−0.30, 0.19)	(−0.28, 0.24)	(−0.28, 0.11)	(−0.09, 0.30)
**Gained**	−0.00	−0.01	−0.06	−0.19
	(−0.02, 0.01)	(−0.14, 0.12)	(−0.17, 0.06)	(−0.49, 0.11)
	**Dropped or Gained 13/8 mmHg**		**Dropped or Gained 13/8 mmHg**	
**Dropped**	−0.17	−0.03	0.21**	−0.05
	(−0.41, 0.06)	(−0.32, 0.26)	(0.02, 0.40)	(−0.13, 0.04)
**No Change**	0.16	0.03	−0.15	0.05
	(−0.06, 0.38)	(−0.26, 0.32)	(−0.38, 0.08)	(−0.04, 0.13)
**Gained**	0.01		−0.06	
	(−0.02, 0.04)		(−0.16, 0.05)	
	**Dropped or Gained 20/13 mmHg**		**Dropped or Gained 20/13 mmHg**	
**Dropped**	−0.17	−0.16	0.18***	.
	(−0.39, 0.04)	(−0.46, 0.14)	(0.05, 0.31)	
**No Change**	0.17	0.16	−0.18***	.
	(−0.04, 0.39)	(−0.14, 0.46)	(−0.31–0.05)	
**Gained**	.	.	.	.

95% Confidence interval in parentheses

* *p* < 0.10

** *p* < 0.05

*** *p* < 0.01

We find statistically significant treatment effects only in women with baseline stage 1 hypertension. Treatment led to a 21pp (95% CI: 0.02-0.40) higher probability of dropping by 13/8mmHg, and an 18pp (95% CI: 0.05–0.31) higher probability of dropping by 20/13 mmHg after adjusting for age, baseline systolic BP, and BMI.

The results from these logistic regressions support providing the treatment specifically to women with baseline stage 1 hypertension. If the treatment were to be provided to 100 women with stage 1 hypertension (all of whom also received free gym memberships, Fitbits, and AYA) we would expect an additional 21 (over a similar group without the food boxes and Fresh Checks) to have a BP drop of 7/5 mmHg. Of these 21, 18 would be expected to drop 20/13 mmHg.

### Effects on quality of life and quality adjusted life years

Changes on the quality of life came from changes in the index scores indicated by the EQ-5D-5 L. For this measure, there was no significant difference between treatment groups in any of the baseline BP categories (see Supplementary Materials S.2). Overall, there was little movement in life-quality index scores, with no treatment or control group from any BP category or for either sex having an average change significantly different from 0.

Given our finding that the change in quality of life did not significantly differ across treatment groups or from 0, we assume no change in quality when estimating change in QALYs. Changes in QALYs therefore came only from changes in life expectancy, which we estimate based on the change in the likelihood of dropping a specified BP threshold with and without treatment.

### Cost-effectiveness analysis

Assumptions about the structure of a potential future implementation of a CHEERS-like protocol and the durability of the effects substantially affect the cost-effectiveness analyses. We present two analyses based on different scenarios of the duration of treatment needed to produce effects. We conduct our analysis upon women with baseline stage 1 hypertension, the group with the strongest statistical evidence of treatment effectiveness. We do not consider other groups because there is no evidence that treatment is effective in those groups, and accordingly treatment will not be cost-effective at any threshold.

Costs associated with CHEERS that we evaluate as relevant to a non-research-based implementation include (1) bank fees associated with delivery of the Fresh Checks (2) text messaging service fees (3) record keeping costs and (4) costs of the food items for the food boxes and the Fresh Checks themselves. Items 1–3 constitute roughly $15 per participant per month, and food boxes plus Fresh Checks were $75 per participant per month. Recruitment costs for this study came to $117 per participant, and include materials such as advertisements and flyers, as well as the time spent with an average hourly wage of $21.05. However, for the purposes of our analyses, we assume a referral-based model which reduces recruitment costs to essentially 0. Thus, in a treatment model that does not require recruitment costs, there is a total of $90 per participant per month to receive the primary CHEERS intervention.

The benchmark we use for the cost-effectiveness of an intervention is $50,000 per QALY, a standard if even perhaps conservative figure [26]. Illustratively, at a cost of $90 per month per participant, if a total of 555.6 months (46.3 years) of CHEERS treatment leads to one gained QALY the treatment is cost-effective.

We analyze the cost-effectiveness of CHEERS under two scenarios. In both scenarios, we consider 100 individuals who receive the treatment for 6 months, as they did in the RCT.

In scenario 1, the treatment ends for all at that point, but we assume durable changes in the BP of recipients that persist after treatment has ceased. Implicitly we are saying that CHEERS initiated a lifestyle change on those for whom it was effective, and they continued to follow CHEERS eating protocol, paying for it themselves. In scenario 2, treatment is continued only for those for whom it has been shown to be effective. We believe this is the more realistic of the two scenarios.

Table 4 shows the expected life years gained from applying CHEERS to 100 individuals as well as the gains in QALYs under the assumption that gains in life expectancy match those suggested by the Sesso et al. study. Based on the effectiveness analysis shown above, for each 100 female participants 18 will show a drop in BP of 20/13 mmHg and 3 will show a drop in BP of 13/8 mmHg. This would lead to a collective gain of 50.79 life-years.

Because we found no difference between treatment groups in the change in life quality, we estimate the QALYs gained as equal to the life-years gained times the utility of each year of life, as extrapolated from the life quality index scores at follow-up for each group. The follow-up average life quality index score for women with baseline stage 1 hypertension was 0.80. The final column in Table 4 adjusts the life-years gained by that utility index, providing the measure for QALYs gained.

**Table 4:** Estimated life years gained by applying CHEERS to target population of 100 individuals with Stage 1 Hypertension using estimates from a 2018–2020 study of hypertension management in Oklahoma

	Additional number expected to drop threshold		Additional life years per individual		Expected life years gained		Expected QALYs gained	
Threshold								
13/8 mmHg	3		1.69		5.07		4.06	
20/13 mmHg	18		2.54		45.72		36.58	
Total Life Years/QALYs Gained					50.79		40.64	

Sesso et al. estimates were used to estimate life-year gains for each attained BP improvement threshold. EQ-5D index scores for women with baseline stage 1 hypertension were on average 0.80

In Table 5 Panel A we present the cost of CHEERS under the two scenarios outlined above. All 100 individuals in both scenarios receive the treatment for first 6 months, at a total cost $54,000 ($90 per month*100 recipients * 6 months). After the initial 6 months, in scenario 2 treatment continues only if it has been effective, throughout the remainder of the lives of the recipients. A National Center for Health Statistics report using 2019 data estimated that life expectancy for 50-year-old Native American women is 30.4 years [27]. We take this estimate to be the baseline, with treatment gains extending this figure. Of the expected 21 women who will continue treatment, 3 will receive it for 32.09 years and 18 will receive it for 32.94 years. This an additional $744,325 and a total cost of $798,325 in scenario 2.

**Table 5:** Costs of implementing CHEERS under different treatment scenarios using estimates from a 2018–2020 study of hypertension management in Oklahoma

**Panel A: Costs of implementing CHEERS per 100 participants**	
Initial 6 months of treatment (Scenario 1 and base for scenario 2)	$54,000
Continued Treatment after 6 months only for responders for duration of life expectancy (Only scenario 2)	$744,325
Total cost of scenario 2	$798,325
**Panel B: Cost for each QALY gained**	
Initial 6 months of treatment	$1,329
Initial 6 months of treatment for 100 participants, then continued treatment after 6 months only for responders for duration of life expectancy	$19,648

Finally, in Table 5 Panel B, we show the cost per QALY gained under the two scenarios. If 6 months of treatment results in lifetime BP improvement without treatment continuing (scenario 1), the cost per QALY gained is an almost miniscule $1,329 per 100 individuals treated. If treatment must be continued to produce the BP reduction (scenario 2), the cost per QALY gained is $19,648, a number which still easily meets the $50,000 threshold.

Because the baseline BP levels used in Sesso et al. are higher than those of women with stage 1 hypertension in our study, we can reasonably expect the actual gains from treatment to be somewhat lower. However, the large gap between the cost per QALY we estimate and the $50,000 cost-effectiveness threshold provides guidance on how attenuated the gains from treatment could be and for treatment to still be cost-effective. If the cost per QALY was 250% higher than our estimate (indicating there is only a small fraction of the experimentally observed benefits) it would still be cost-effective to provide the treatment to women with stage 1 hypertension.

## Discussion

In this study, we examine the efficacy of a monthly healthy food box in hypertensive patients in combination with gym memberships, Fitbits, and a culturally tailored walking app (AYA). Our analysis suggests that a CHEERS-type treatment has benefits for women with baseline stage 1 hypertension. For this group, adding food boxes and Fresh Checks to AYA and Fitbits significantly improved the probability that individual BPs would fall by thresholds that improve life expectancy. We find that the treatment meets a normative cost-effectiveness threshold of $50,000 per QALY even in the case that treatment must be provided for the remaining life of recipients for whom treatment is effective.

While we found treatment improved estimated life expectancy, we found no evidence that this treatment improved the quality of life as measured by the EQ-5D. Despite any improved health that comes with better control of hypertension, there was no significant improvement from treatment in the life-utility index we used for this study. One note is that the EQ-5D may not be capturing all of the quality-of-life changes that could be expected from long-term BP levels; a stroke or heart failure, for instance, would lead to a reduced quality of life.

We additionally note that there may be non-dietary mechanisms linking treatment with improvements in BP. It may be that receiving treatment improved utilization of or focus on the other resources like the gym or Fitbit. This does not detract, however, from our conclusion that healthy food box delivery and fresh checks yield benefits in combination with those resources.

While we did not find significant treatment efficacy in 3 of the 4 subgroups examined, one of the primary lessons of our study is that the relatively low cost of a nutrition-based treatment means that such treatments can be cost-effective if targeted towards certain groups. While in this manuscript we do not examine mechanisms which could explain why treatment was only effective in women with stage 1 hypertension, our results suggest that further research on expanding the groups for whom the CHEERS intervention is effective is warranted. Using larger samples will be important for stronger identification of differences in treatment effects between subgroups.

The limits of our conclusions are many. First, the treatment was applied to a very specific population, the Chickasaw Nation in Oklahoma. Moreover, within our study the treatment was provided along with access to a free tribal gym, a Fitbit, and AYA. These latter three benefits were also provided to the control group. Hence, our analysis is of the benefits of the food boxes and Fresh Checks, but only for a population that also has these additional instruments that might help control BP. It may be that the treatment works only if these additional resources are available. And it is important to note that the treatment was in addition to, not in substitution for, care that participants were already receiving. Finally, the most serious limitation of this study was its small sample size, which led to even smaller comparison groups in our disaggregated analysis and corresponding low power to detect treatment effects, particularly in men. A larger study will be necessary to generate more reliable estimates of treatment effects, and the results presented here should be interpreted with caution.

## Conclusion

Directly providing hypertension patients with DASH foods overcomes some of the key behavioral barriers to adhering to a DASH diet. We demonstrated in a group randomized controlled trial that a nutrition-based treatment desirable for other reasons appears to also be effective and cost-effective for Native American women with stage 1 hypertension, although our sample size was small, and a larger study will be necessary to build upon our results. Further research is warranted to understand why we found an impact only for this group.

### Electronic supplementary material

Below is the link to the electronic supplementary material.

Supplementary Material 1

## Data Availability

Data available upon reasonable request.
