# Supplementary material for: The cost-efficacy of a healthy food box for managing hypertension within a native American population: a group randomized controlled trial
Source: Arch Public Health. 2024 Apr 26;82:59. doi: 10.1186/s13690-024-01274-9 (PMC11046939; doi:10.1186/s13690-024-01274-9)
Supplement: Supplementary file 1 — Supplementary Material 1 [file 13690_2024_1274_MOESM1_ESM.docx]

# Supplementary Materials

## S.1 Additional details on the intervention

Food box contents were designed by a registered dietician to be DASH-approved and culturally appropriate, provided by the tribally-run Packed Promise for a Healthy Heart program, and packed at and shipped by the Regional Food Bank of Oklahoma.

These boxes contain shelf-stable, DASH-approved items for preparing low sodium, low fat meals, tailored for hypertensive individuals. Food items varied each month, however, food servings provided in the boxes were standardized to provide participants with a 30-day supply of the following: 1 serving of fruit (fresh, dried, or canned)/day, 1 serving of vegetable (fresh or low sodium canned)/day, 1 serving of unsalted nuts or seeds/day, 1 serving of beans or lentils/day, and 2 servings of fatty fish (canned)/week. Participants also received a new Ms. Dash seasoning each month (lemon pepper, chipotle, garlic herb, onion and herb, original and table blend).

Fresh checks were redeemable for fresh or frozen fruit and vegetables at Oklahoma Woman, Infants & Children program approved grocery stores within the Chickasaw Nation or at Chickasaw Nation approved local Farmers’ Markets.

## S.2 Quality of life

Table S.1 shows the average change in EQ5D utility index by sex, baseline hypertension category, for both treatment and control groups. In no case was there a statistically significant difference between the groups.

**Table S.1**: Average change in EQ5D index among participants of a group randomized controlled trial on hypertension management in Oklahoma from 2018-2020 by treatment, baseline hypertension category, and sex

| **Baseline blood pressure category** | **Control** | | **Treatment** | | **p-value** |
| --- | --- | --- | --- | --- | --- |
| **Pooled** |  |  |  |  |  |
| Normal | 0.1 | (0.1) | 0.1 | (0.1) | 0.86 |
| Elevated | -0.1 | (0.2) | 0.0 | (0.2) | 0.53 |
| Stage 1 hypertension | -0.1 | (0.2) | -0.0 | (0.2) | 0.78 |
| Stage 2 hypertension | -0.0 | (0.1) | 0.0 | (0.1) | 0.41 |
|  |  |  |  |  |  |
| **Female** |  |  |  |  |  |
| Normal | 0.1 | (0.1) | 0.1 | (0.1) | 0.87 |
| Elevated | 0.0 | (.) | -0.0 | (0.3) | . |
| Stage 1 hypertension | -0.0 | (0.1) | -0.1 | (0.2) | 0.61 |
| Stage 2 hypertension | -0.0 | (0.1) | -0.0 | (0.2) | 0.82 |
|  |  |  |  |  |  |
| **Male** |  |  |  |  |  |
| Normal | . | . | 0.1 | (0.1) | . |
| Elevated | -0.1 | (0.2) | 0.1 | (0.2) | 0.24 |
| Stage 1 hypertension | -0.1 | (0.3) | -0.0 | (0.2) | 0.31 |
| Stage 2 hypertension | -0.0 | (0.2) | 0.0 | (0.1) | 0.18 |

*P-value from 2-sided t-test, standard error in parentheses.*
